# Effects of an eastward shift in the Agulhas retroflection region on chlorophyll-a bloom on its south flank

**DOI:** 10.1371/journal.pone.0281766

**Published:** 2023-03-27

**Authors:** Xiaoqi Ding, Hao Shen, Jialong Sun, Linfei Bai, Lin Peng, Haibin LÜ

**Affiliations:** 1 Jiangsu Key Laboratory of Marine Bioresources and Environment /Jiangsu Key Laboratory of Marine Biotechnology, Jiangsu Ocean University, Lianyungang, Jiangsu Province, China; 2 Lianyungang Meteorological Bureau, Lianyungang, Jiangsu Province, China; 3 School of Marine Technology and Geomatics, Jiangsu Ocean University, Lianyungang, Jiangsu Province, China; 4 Co-Innovation Center of Jiangsu Marine Bio-industry Technology, Jiangsu Ocean University, Lianyungang, Jiangsu Province, China; Second Institute of Oceanography Ministry of Natural Resources, CHINA

## Abstract

As observed by remote sensing images in December 2013 and January 2014, chlorophyll-a (Chl-a) bloom occurred on the south side of the Agulhas Current (38°S-45°S). The dynamic mechanisms of Chl-a bloom were studied by satellite remote sensing data, reanalysis data and Argo data. The periodic shedding of the Agulhas ring led to a significant eastward shift of the Agulhas retroflection from December 2013 to January 2014, without the obstruction of flowing complex eddies and with increased current flow. Then, the horizontal transfer of Chl-a occurred along the south side of the Agulhas Current (38°S-45°S). Nitrate concentrations reached 10–15 μmol·L^-1^ on the south side of the Agulhas Current, where a deepened mixed layer and upwelling and the vertical transport of nutrients contributed to the Chl-a bloom. In addition, sufficient light and suitable precipitation provide good conditions for Chl-a bloom on the south side of the Agulhas Current.

## Introduction

The Agulhas Current moves southward along the Indian Ocean near South Africa in the form of the Western Boundary Current. The current has a velocity of over 2 m·s^-1^ and carries heat and nutrients from the equatorial Indian Ocean to subtropical latitudes [1,2]. It is a key component of global ocean circulation, and its change will have a significant impact on the surrounding ocean environment and even the global climate [1,3].

Agulhas Current splits into two branches after passing the Agulhas bank [4]. One branch, the so-called Agulhas Return Current. The Agulhas Current carries South Indian Subtropical Surface Water, Antarctic Intermediate water, some remnants of Red Sea Water and tropical Indian surface water into the Agulhas retroflection region [5]. At the this loop, known as the Agulhas Retroflection, the Agulhas Current turns back on itself, where the ring formed in this reversal region is the largest mesoscale eddy in the world [6]. The Agulhas Retroflection sheds rings, eddies and filaments to the west, where the impact depth is greater than 2,000 m [3]. Most of its waters subsequently flow eastward as the Agulhas Return Current, while the Agulhas ring carrying its load of South Indian water masses will drift off into the South Atlantic Ocean, creating Agulhas leakage. The Agulhas leakage region is characterized by vigorous variability on intraseasonal to interannual timescales [6]. The main controlling factors on retroflection and leakage are the latitude of maximum westerlies and the southward inertia of the Agulhas Current at the separation time, which are both largely determined by the strength and position of the wind field over the Indian Ocean [3]. The strong Agulhas Return Current and the complex front system (including the Agulhas Return Front, the Subtropical Front and the Subantarctic Front) make the Southwest Indian Ocean one of the most energetic regions across the global oceans [7], which then have significant impacts on phytoplankton production [8].

Based on investigations spanning a period of 10 years, it was shown that Agulhas rings may be present at the location of the core approximately 12% of the time [5]. Van Aken et al. reported that there is a significant eastward contraction of the Agulhas retroflection region after the loss of three rings of the Agulhas Current [9]. Some researchers have speculated that climate change may cause the shedding of rings, although this is still a guess [10]. While the eddy shedding process does not show any obvious seasonality, it might not be so for the Agulhas Current retroflection itself. A seasonal cycle of the retroflection does exist, the retroflection was observed further west in summer and further east in winter [11,12]. Dencausse et al. observed that from October 1992 to the end of 1997, the retroflection was clearly shifted westward during the austral summer and eastward during winter [12]. Van Aken et al. reported that in October 2000, the Agulhas Current moved eastward along the slope of the Agulhas bank, the eastward shift of the retroflection in November, the further retraction of the retroflection to the Transkei in December, and from February to April 2001 moved westward, and in May, the Agulhas Current again moved eastward along the slope of the Agulhas retroflection region [9].

Some research show that nonlinear mesoscale eddies can affect the biogeochemical cycling of the upper ocean through vertical and horizontal advection of nutrients and marine organisms [13]. The large enrichment of Chl-a in December 2013 and January 2014 in remote sensing images occurred when there was a significant eastward shift in the Agulhas retroflection region. Based on several studies, it is known that the causes of Chl-a bloom are diverse [13–15]. Is there a relationship between the Chl-a bloom event and the eastward shift of the Agulhas Current retroflection? Can the southward transfer of Chl-a increase due to the eastward shift of the Agulhas Current retroflection? Up to date, it seems that little research has been done on these issues.

In this study, we analyzed the dynamic mechanisms of Chl-a bloom on the southern side of the Agulhas Current (38°S-45°S) in December 2013 and January 2014. The data and methodology are presented in Section 2, which is followed by the results in Section 3. Sections 4 and 5 present the discussion and conclusion, respectively.

## Data and methods

### Study area

**Fig 1:**
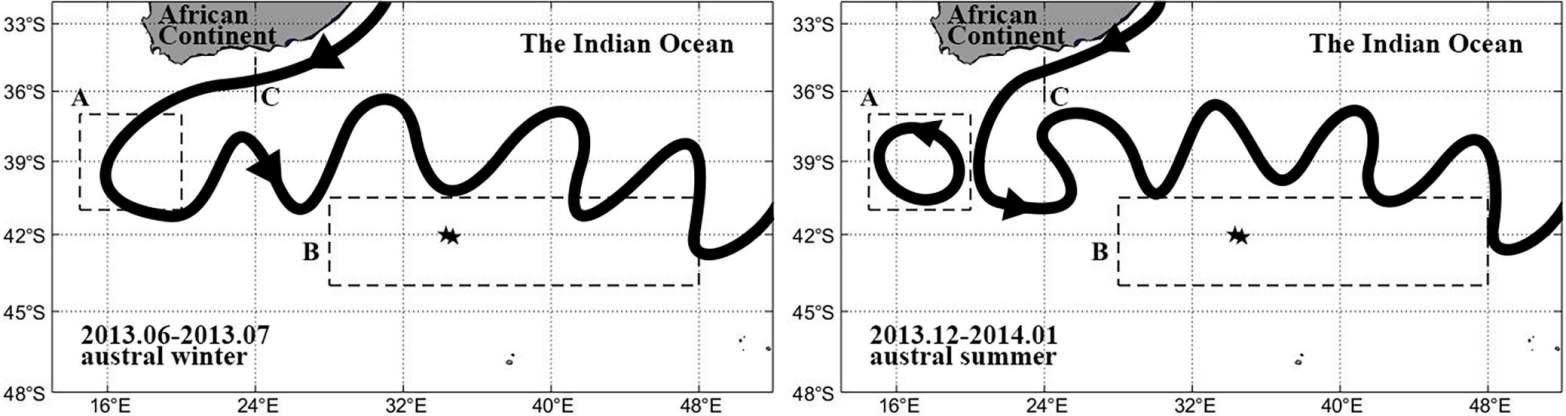
The general flow direction of Agulhas Current in winter and summer. Box A is at the Agulhas retroflection region. Box B is located at the south of the Agulhas Current. Line C is at the point where the Agulhas Current out of the African continent before entering the retroflection region. The black pentagrams are the location of the Argo.

In the study, the general flow directions of Agulhas Current in winter and summer are shown in Fig 1 and two boxes and one section were selected. Box A (37°S-41°S, 14.5°E-20°E) is located in the Agulhas retroflection region for observing the eastward shift and Chl-a horizontal transfer phenomenon. Box B (40.5°S-44°S, 28°E-48°E) is located on the south side of the Agulhas Current for observing the Chl-a bloom phenomenon. Section C (34.5°S-36.5°S, 24°E) was used to observe the seasonal variation in the Agulhas Current. In Table 1, the average Chl-a concentrations in Box B during austral winter and summer from 2010 to 2015 and whether the eastward shift of the retroflection region occurred were listed. It can be seen that the Chl-a bloom was much higher in austral summer than in winter during all 6 years. This indicates that summer provides superior conditions for Chl-a bloom. However, in 2011 and 2013, the amount of Chl-a bloom in austral summer was higher than in the other years of the same period, reaching more than 0.1mg·m^-3^. It shows that the eastward shift of the retroflection region occurred in 2010 and 2013, so it is reasonable to speculate that the Chl-a bloom that occurred on the south side of the Agulhas Current was influenced by the eastward shift of the retroflection region in addition to the superior Chl-a growth conditions in austral summer. June and July 2013, December 2013 and January 2014 were chosen as a typical representative time period for study.

**Table 1:** Observation of Chl-a concentration and shifting eastward retroflection from 2010 to 2015.

Year	Average Chl-a concentrations in Box B		The higher Chl-a concentrations	Retroflection region shifted east
	June-July (mg·m^-3^)	December-next January (mg·m^-3^)		
2010	0.027051455	0.067341734	austral summer	No
2011	0.014871276	0.105222368	austral summer	Yes
2012	0.018560967	0.079428208	austral summer	No
2013	0.028101624	0.120731736	austral summer	Yes
2014	0.012618812	0.091827722	austral summer	No
2015	0.010473335	0.085661731	austral summer	No

### Data

Daily Chl-a and photosynthetically available radiation (PAR) with a 4 km spatial resolution derived from the GlobColor L3 product (http://hermes.acri.fr/index.php?class=archive). The GlobColor project started in 2005 as an ESA Data User Element (DUE) project to provide a continuous dataset of merged L3 Ocean Color products. The GlobColor dataset provides a large set of merged ocean color products. Except for the Chl-a data used in the calculation of Chl-a flux, all the rendering of Chl-al remote sensing images comes from this website. This product was also used in the study of Tan et al. [14] and Lu et al. [15].

Since Chl-a and flow field data with the same resolution are required to calculate flux, all data for calculated flux are derived from reanalysis data. Such as the direction and flow of ocean currents, were all obtained from the CMEMS project (https://resources.marine.copernicus.eu/products). The project’s data is also being used by Xia et al. [16] and Lü et al. [17] in their study.

Nitrate and phosphate concentrations at 1°×1° resolution are available from the World Ocean Atlas (WOA2013, https://www.ncei.noaa.gov/data/oceans/woa/WOA13/DATAv2/), from which the nitrate data in December 2013 were taken. The World Ocean Atlas (WOA) is a collection of objectively analyzed, quality controlled temperature, salinity, oxygen, phosphate, silicate, and nitrate means based on profile data from the World Ocean Database (WOD). Climatology mean of nitrate concentrations from the same source had also been utilized in the study of Chl-a [15,18].

Precipitation data for June, July and December 2013 and January 2014 were provided by product 3B42. The product was provided by the Tropical Rainfall Measuring Mission project (TRMM: https://daac.gsfc.nasa.gov/), which is three-hour precipitation data with a spatial resolution of 0.25°×0.25°.

Argo buoys can detect and record temperature and salinity data within 100 m below the ocean surface. This study utilizes Argo platform numbers 1901371, 3901015 and 3900762, which are indicated by the diamond, quadrilateral and hexagon in Fig 1. The platform data were from the Indian Argo project (https://dataselection.euro-argo.eu/).

### Methods

#### Vorticity

The calculation of the curl of the sea current vector (u, v) can be similar to that of Lu et al. [15]:

curl=(dv/dx)−(du/dy) (1)

where *u* and *v* are the two velocity components along the *x* and *y* directions.

Brunt–Vaisala frequency. In studies of atmospheric dynamics and oceanography, to measure the stability of fluids caused by convection, the Brunt–Vaisala frequency (N) is generally utilized, which can be calculated as

$$N=\sqrt{\frac{-g}{\rho }\times \frac{d(\rho )}{d(z)}}$$ (2)

where *ρ* is the potential density, *g* is the local acceleration of gravity, and z is the geometric height. The maximum Brunt-Vaisala frequency can be defined as the location of the thermocline [15].

## Results

### Variation in Chl-a concentration

**Fig 2:**
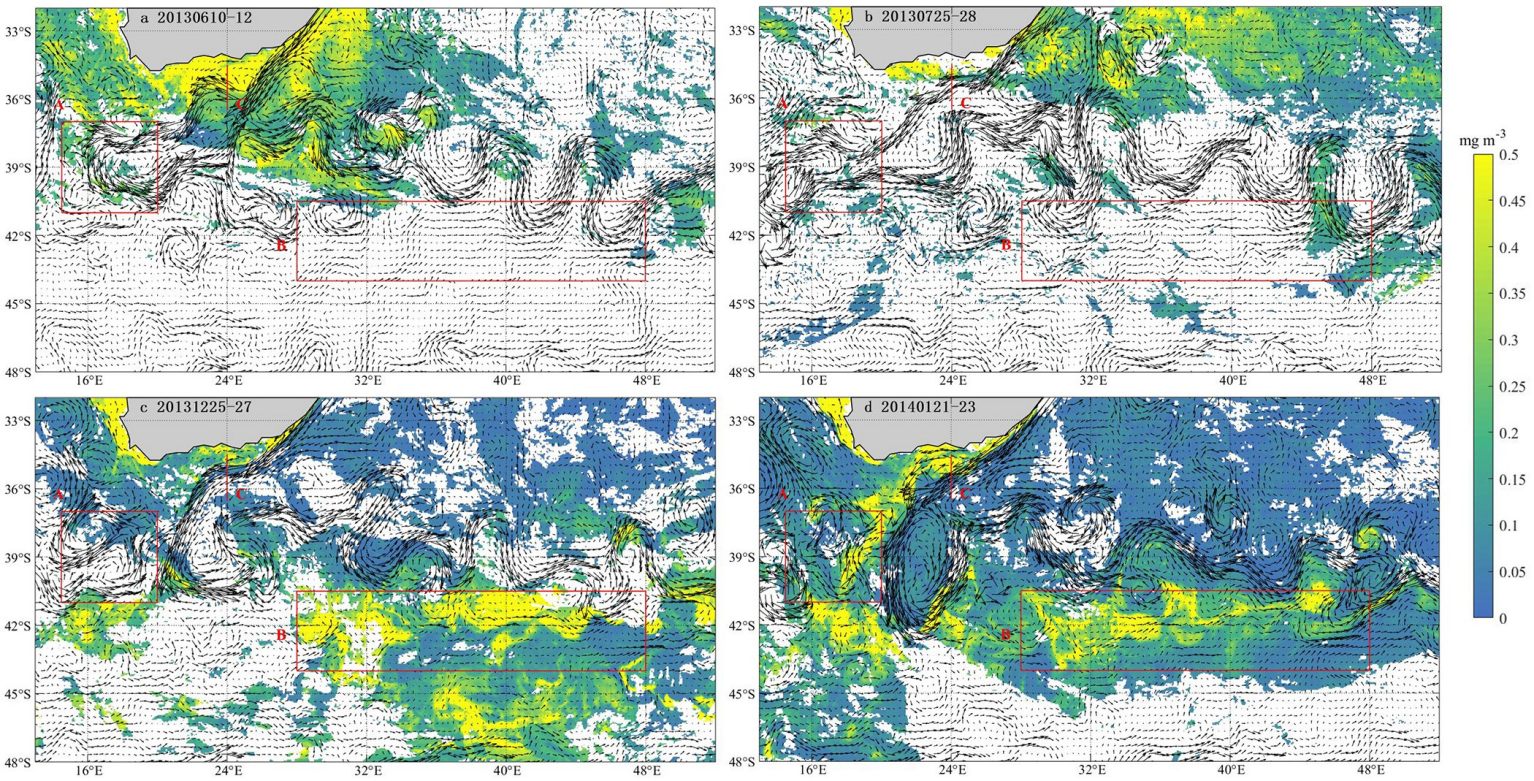
The 3-day average of the flow field and Chl-a. (a) 2013.6.10–12, (b) 2013.7.25–27, (c) 2013.12.25–17, (d) 2014.1.21–23. Color bars denote Chl-a concentration (unit: mg·m^-3^) and black arrows indicate the direction of the Agulhas Current.

To observe the changes in Chl-a around the Agulhas Current, data from June and July 2013(austral winter), December 2013, and January 2014(austral summer) were selected at 13°E-52°E and 32°S-48°S. The 3-day average of the flow field and Chl-a in the study area are shown in Fig 2.

**Fig 3:**
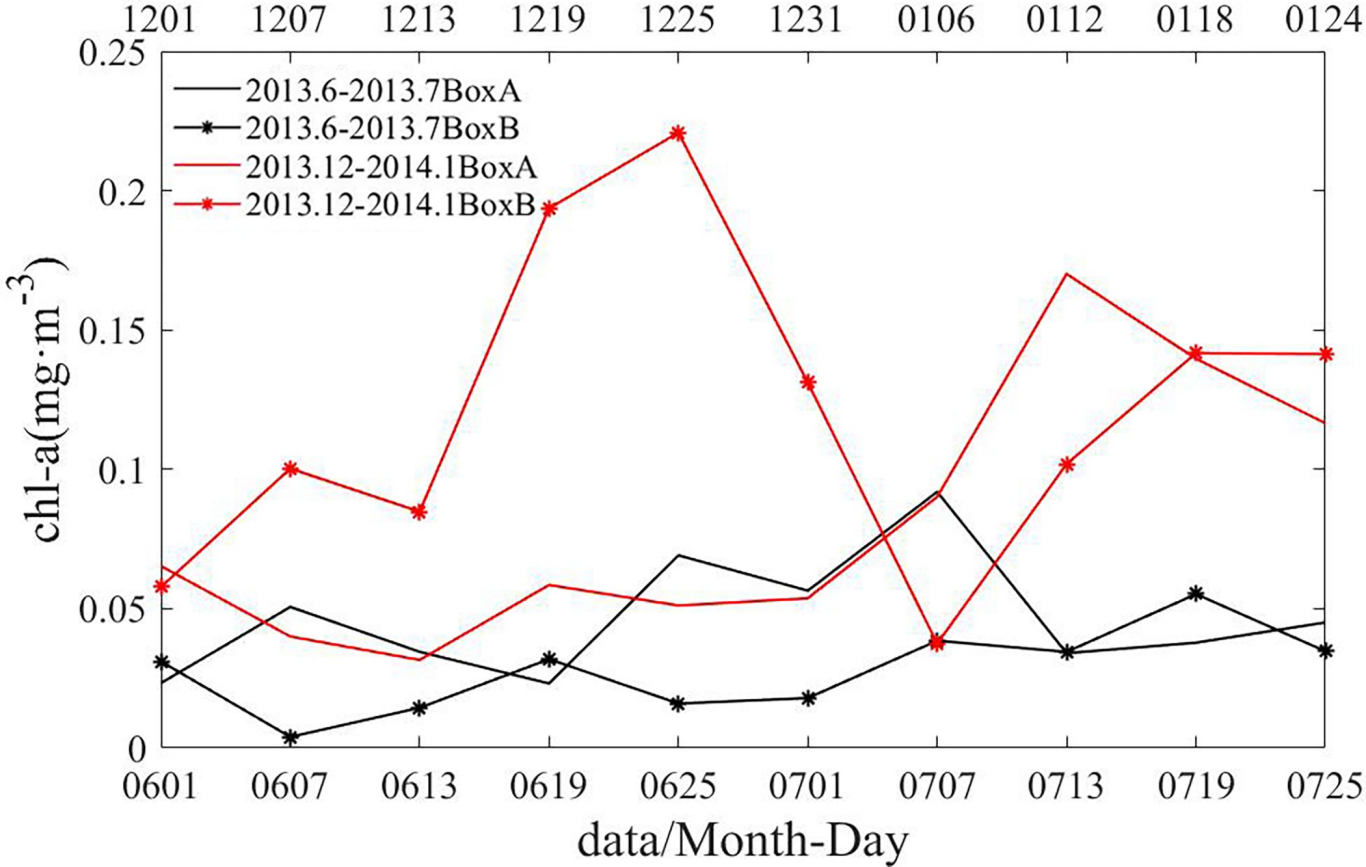
Chl-a concentration in Boxes A and B in June and July 2013 (black line) and December 2013 and January 2014 (red line).

Chl-a on the south side of the Agulhas Current after the retroflection has an obvious bloom phenomenon in December 2013 and January 2014. The surface Chl-a concentrations in Box A and Box B are shown in Fig 3. In Box B, the maximum concentration of surface Chl-a in December 2013 and January 2014 reached 0.23 mg·m^-3^, which was much higher than the 0.06 mg·m^-3^ in June and July 2013. In Box A, which was in the retroflection region, the maximum concentration of surface Chl-a in December 2013 and January 2014 reached 0.17 mg·m^-3^, which was higher than the 0.09 mg·m^-3^ in June and July 2013.

### The eastward movement of the Agulhas retroflection region

**Fig 4:**
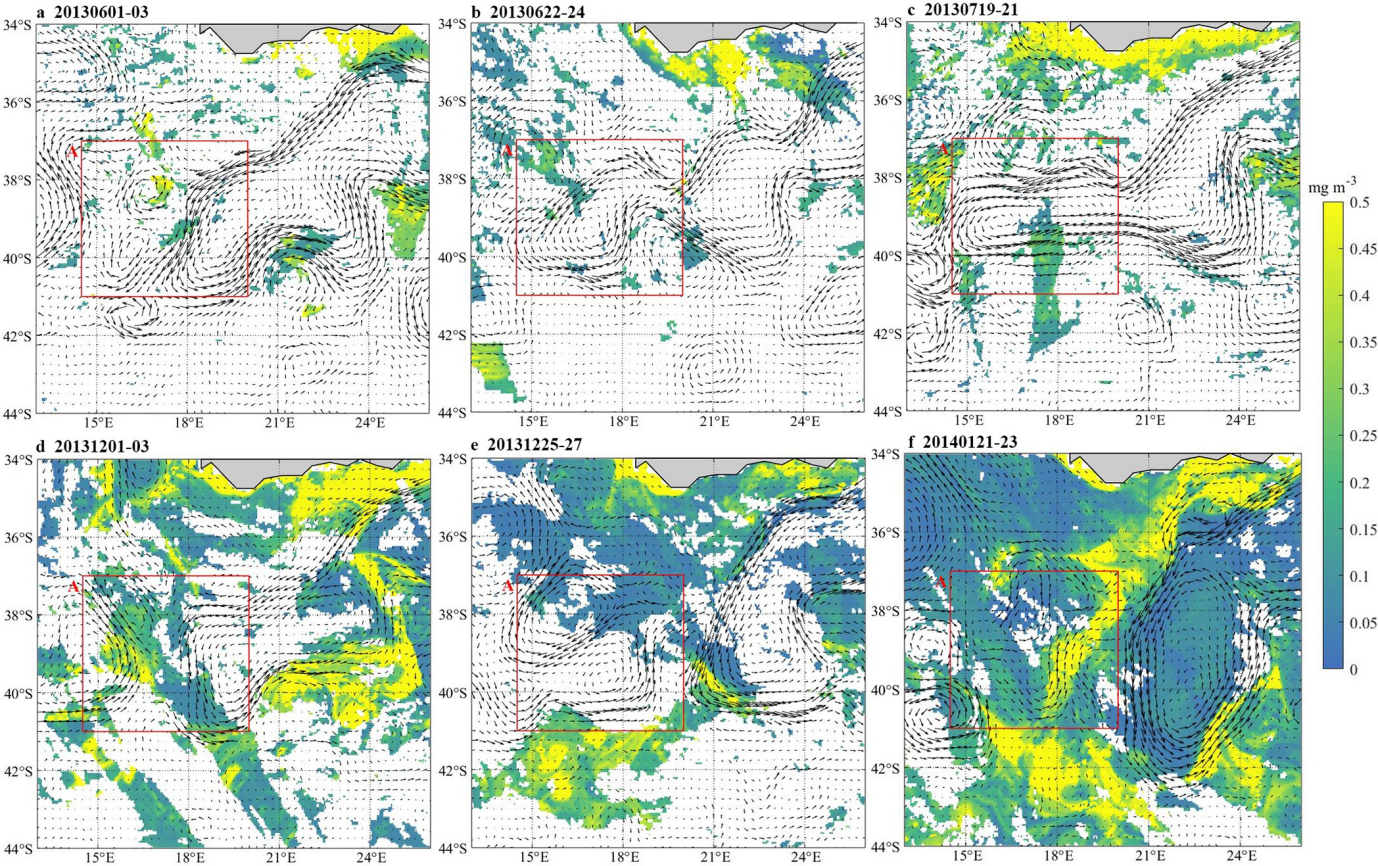
The 3-day average of surface Chl-a and flow field around Box A on 2013.6.1-3(a), 2013.2.22-24(b), 2013.7.19-21(c), 2013.12.1-3(d), 2013.12.25-27(e), and 2014.1.21-23(f). Color bars denote Chl-a concentration (unit: mg·m^-3^) and black arrows indicate the direction of the Agulhas Current.

The 3-day average of surface Chl-a and the flow field around box A in June and July 2013, December 2013 and January 2014 are shown in Fig 4. Box A is located in the Agulhas retroflection region, which can encircle the entire Agulhas ring that is about to fall off. In December 2013 and January 2014, the eastern boundary of Box A is tangent to the Agulhas retroflection. What can be clearly seen is the eastward shift of the Agulhas retroflection region during December 2013 and January 2014. Relative to June and July 2013, Chl-a in box A increased significantly and spread from northeast to southeast (Fig 4F).

**Fig 5:**
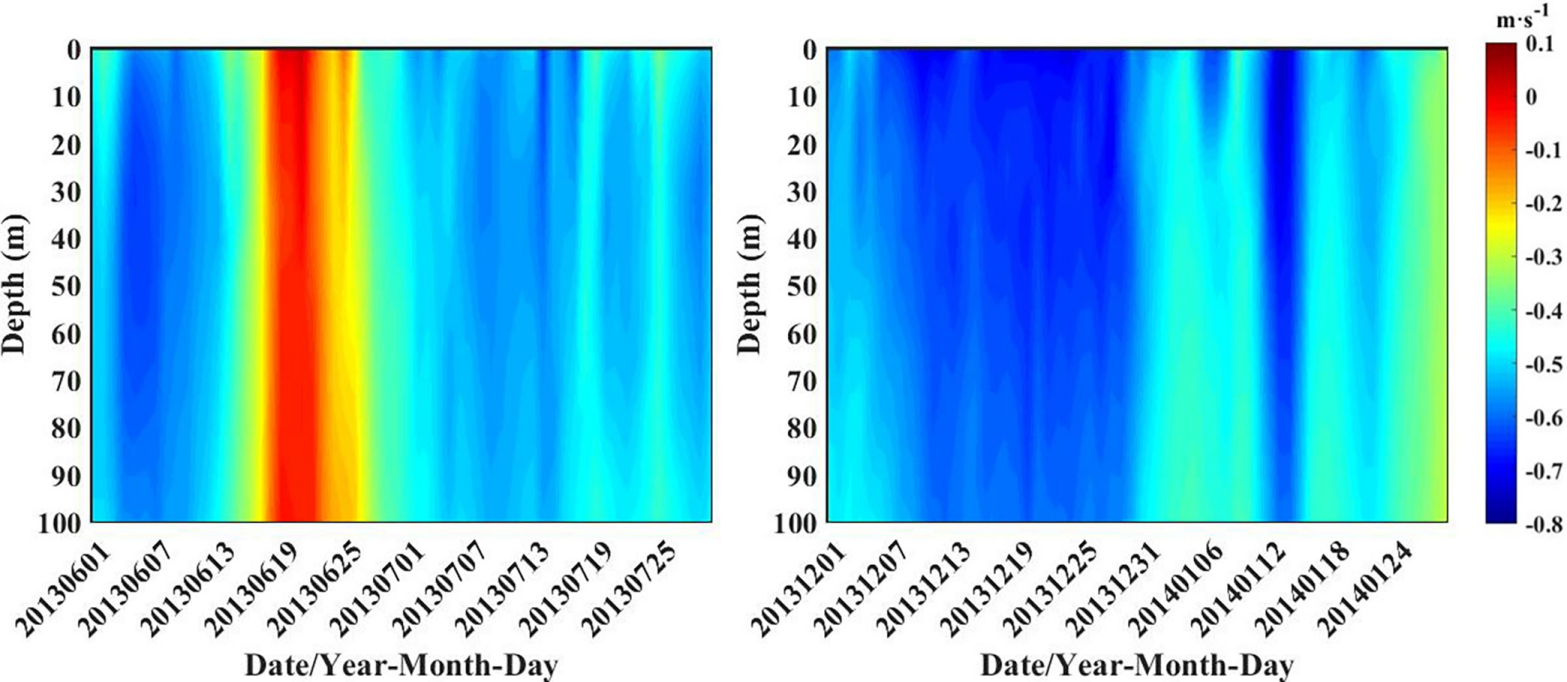
The flow rate of the Agulhas Current through line C.

The flow rate of the Agulhas Current through line C is shown in Fig 5. Positive values represent eastward flow, and negative values represent westward flow. Although the flow trend in all four months is westward, the flow trend in December 2013 and January 2014 is relatively large. Flow velocities were greater than 0.4 m·s^-1^ throughout December, especially in the surface layer, where they could reach 0.8 m·s^-1^. In contrast, flow velocities in June and July were less than that in December 2013 and January 2014. Therefore, the horizontal transports in December 2013 and January 2014 are stronger.

### Chl-a flux

**Fig 6:**
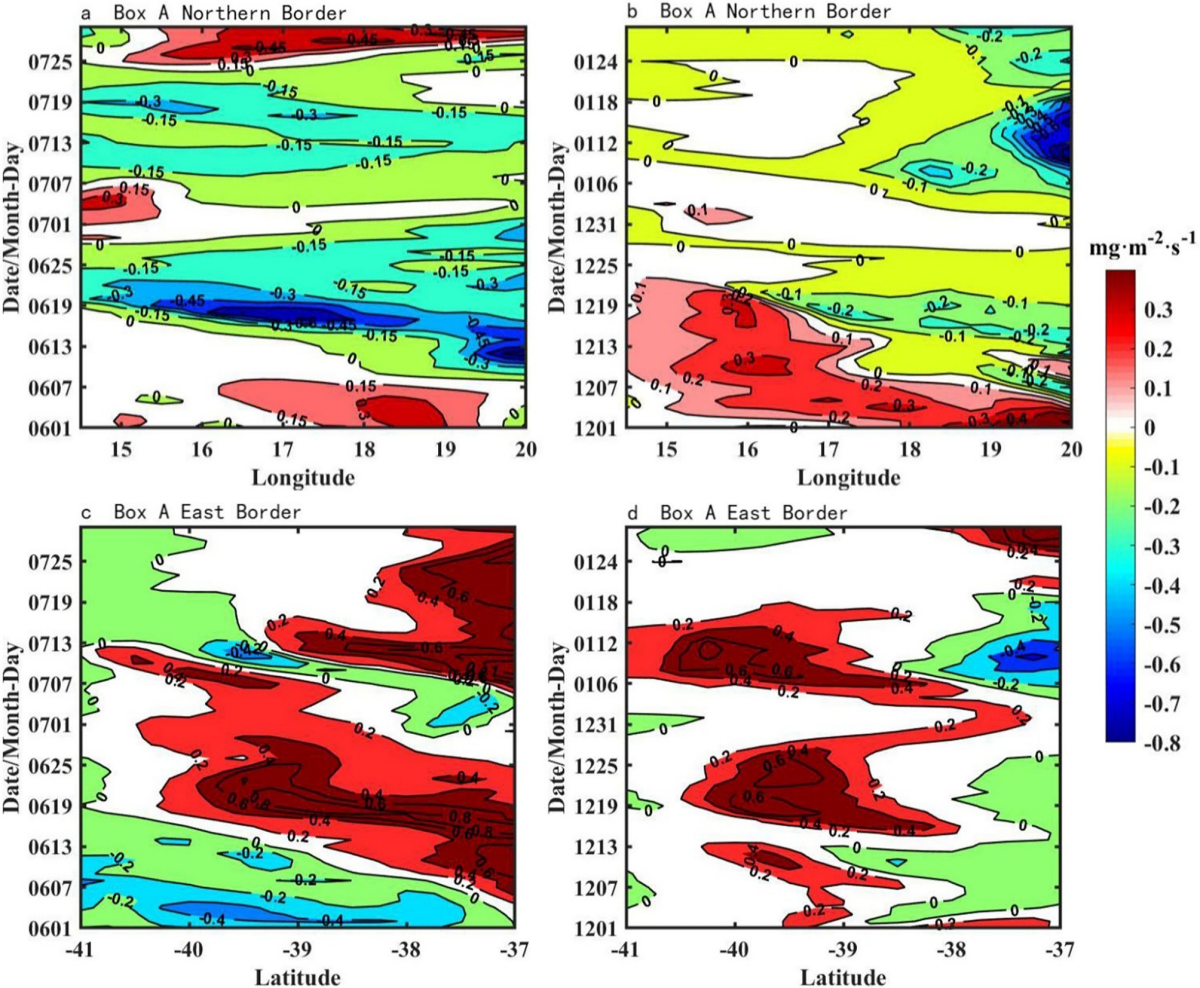
Time series of Chl-a flux in Box A (a) north (c) east boundary from June to July in 2013, (b) north (d) east boundary from December 2013 to January 2014.

**Fig 7:**
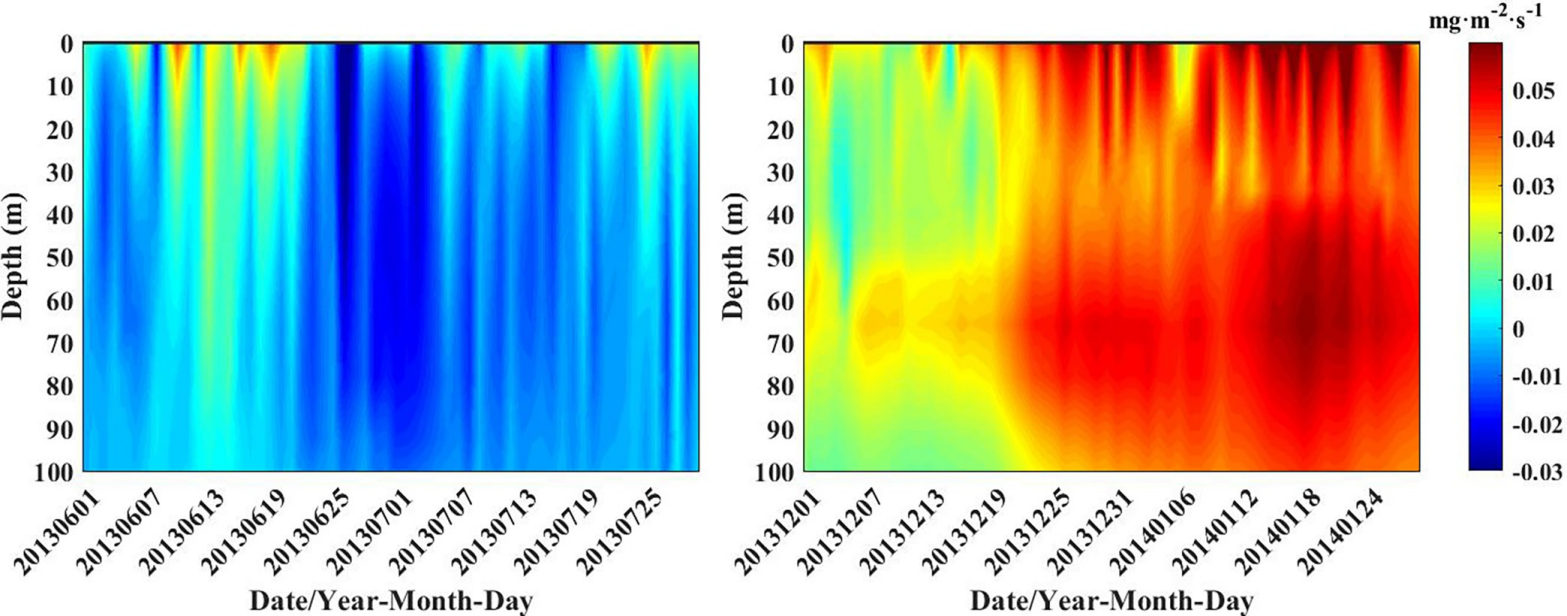
Time series of vertical distributions of Chl-a flux at the western boundary of Box B.

The direction of horizontal Chl-a transfer can be observed in Fig 4. Chl-a fluxes flow in (out) Box A from the northeast (south) boundary of Box A and are then transported along the south side of the Agulhas Current. To confirm the horizontal transfer direction of surface Chl-a more clearly, the Chl-a flux figures were drawn (Figs 6 and 7). Using the Chl-a concentrations multiplied by the longitudinal velocity v gives the Chl-a fluxes on the south and north sides. Similarly, the Chl-a fluxes on the east and west sides are obtained by performing the same operation with the lateral velocity u and the Chl-a concentrations. A negative value for the north and east sides indicates an inflow of Chl-al, while the opposite is true for an outflow. On the south and west sides, positive values indicate inflow, and negative values indicate outflow [16].

As seen from the four pictures of Fig 6, the inflow of Chl-a in December 2013 and January 2014 was concentrated at 18°E-20°E at the northern border and at 37°S-38°S at the eastern border compared to June and July 2013, and the Chl-a inflow was from the northeastern direction of Box A. The Chl-a flux at the western boundary of Box B in June and July 2013, December 2013 and January 2014 is shown in Fig 7. In December 2013 and January 2014, large amounts of Chl-a flowed in from the western border of Box B, with concentrations between 0.06 mg·m^-2^·s^-1^ and 0.08 mg·m^-2^·s^-1^. In contrast, in June and July 2013, the inflow from the western boundary of Box B reached less than 0.02 mg·m^-2^·s^-1^. From Figs 6 and 7, it can be found that the path of Chl-a horizontal transport passed from the northeast to the southeast of Box A and finally transports eastward into Box B.

### Climatology nitrate and phosphate distribution around the Agulhas Current

**Fig 8:**
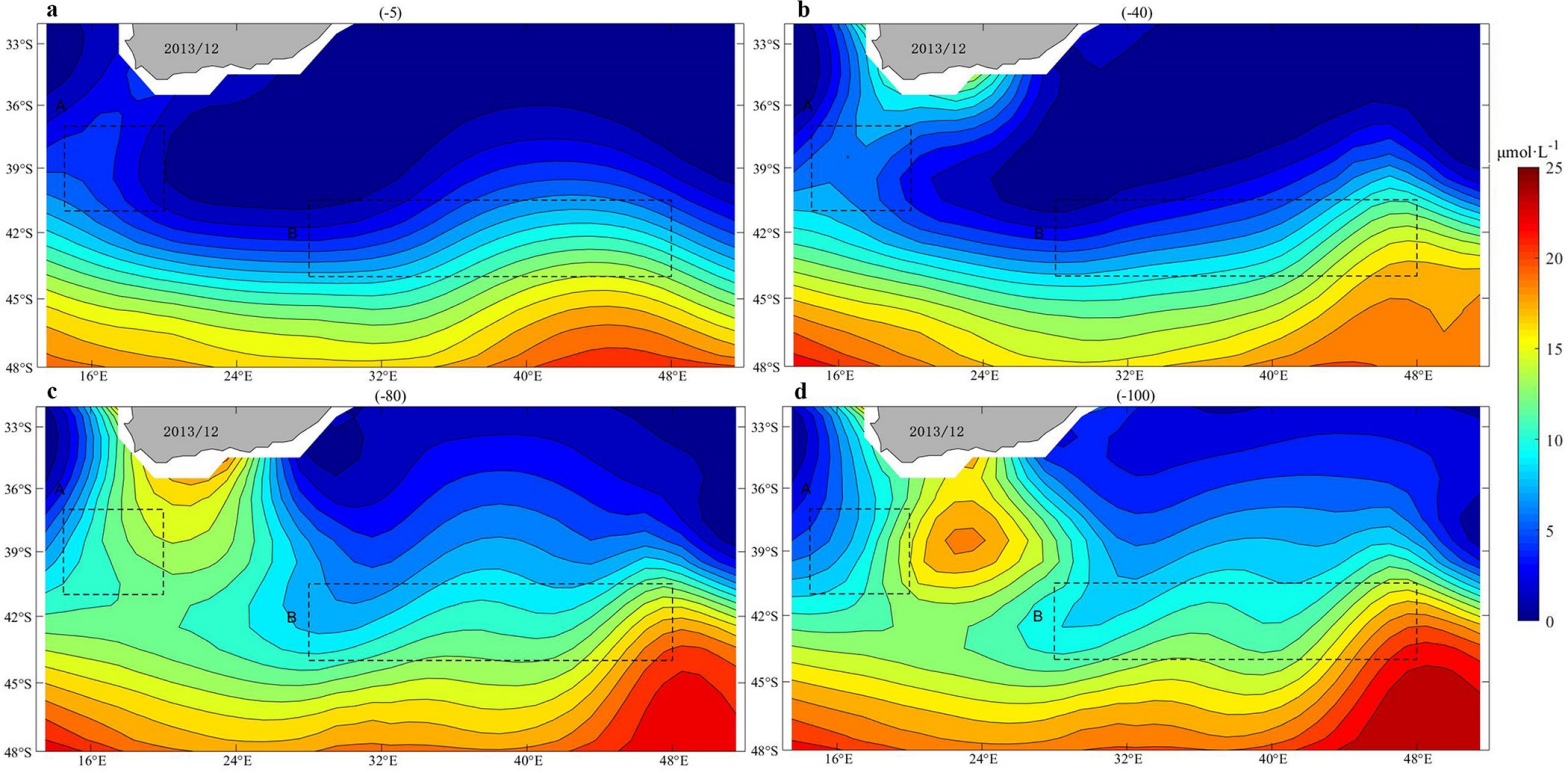
Climatology mean of nitrate concentrations (μmol·L-1) in December (the results at depths of -5 m, -40 m, -80 m and -100 m, respectively).

**Fig 9:**
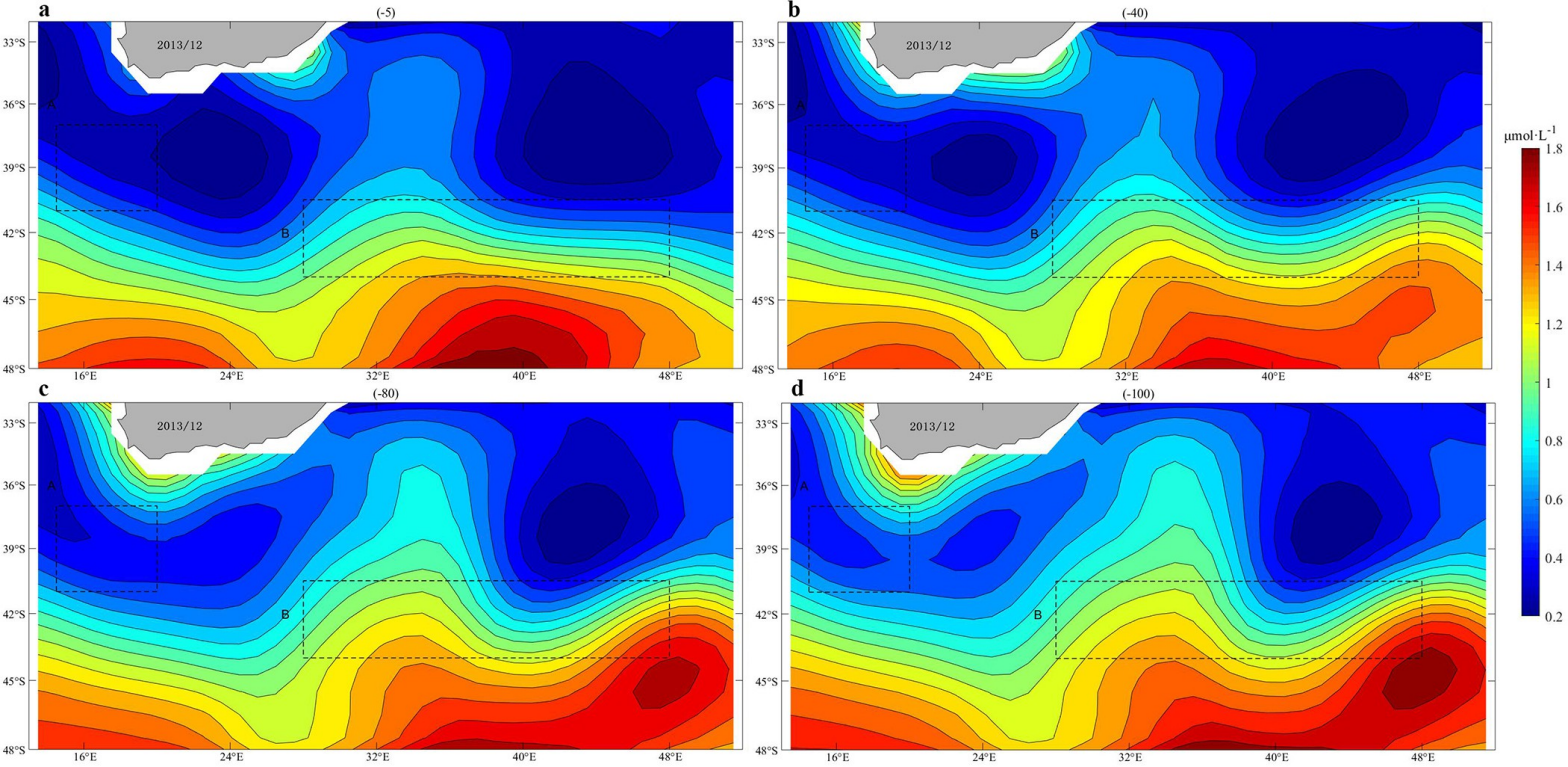
Climatology mean of phosphate concentrations (μmol·L-1) in December (the results at depths of -5 m, -40 m, -80 m and -100 m, respectively).

Both phosphate and nitrate are important nutrients for phytoplankton growth. The high concentrations of nitrate and phosphate were mainly distributed on the south side of the Agulhas Current, as shown in Figs 8 and 9. Nitrate concentrations on the southern side of the Agulhas Current were concentrated at 10–15 μmol·L^-1^, especially at depths of 80 and 100 m. Compared to the north side, the content was between 0–5 μmol·L^-1^. Phosphate concentrations reached 1.2–1.8 μmol·L^-1^, at all depths, higher on the south side than that on the north side. In subtropical circulation, phytoplankton growth is mainly limited by nutrient availability [19,20]. Nutrient-rich upwelling can lead to aggregation of phytoplankton in the upper layers [18,21].

### Photosynthetic effective radiation (PAR)

The time series of spatially averaged PAR in Box B in June and July 2013, December 2013 and January 2014 are shown in Fig 9. In Box B, PAR was above 40 Einstein m^2^·day^-1^ in both December 2013 and January 2014, while it was below 10 Einstein m^2^·day^-1^ in all of June and July 2013. A high amount of sunlight enters the euphotic layer [15], which may be a significant influencing factor for the Chl-a bloom event on the south side of the Agulhas Current. Similar conclusion was also reached by Gu et al., in their study on the relationship between Chl-a and mixed layer depth, where Chl-a might be limited by light in spring-autumn-winter and by the nutrient in summer [8]. Swart et al. have also suggested that the upper ocean stratification can induce springtime bloom by increasing the mean light level for the phytoplankton [22].

### Precipitation

**Fig 10:**
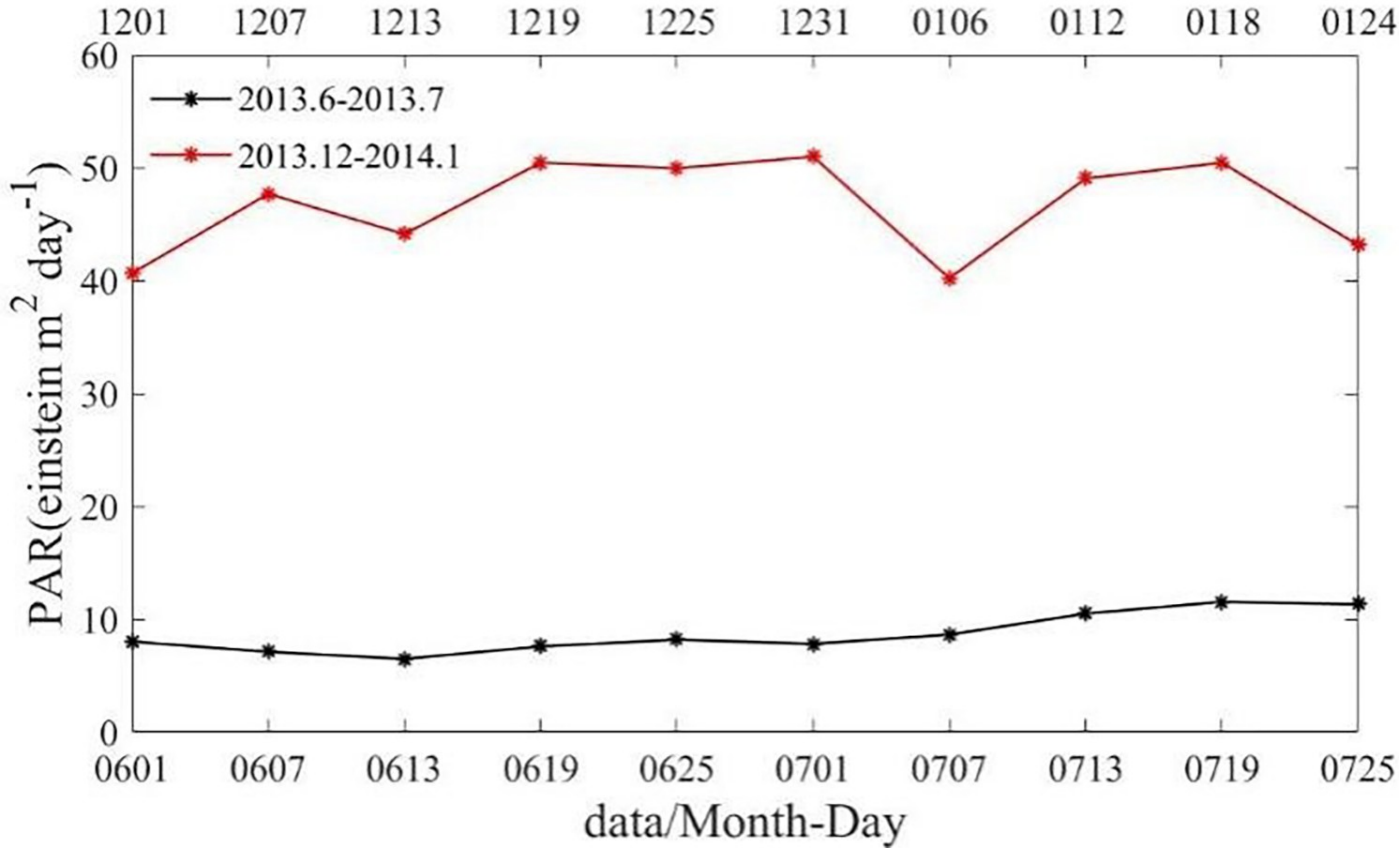
Time series of spatially averaged PAR in Box B in June and July 2013 (black line), December 2013 and January 2014 (red line).

As shown in Fig 10, in Box B, the average precipitation in December 2013 and January 2014 was approximately 10 mm·day^-1^, while the average precipitation in June and July 2013 was twice as high 20 mm·day^-1^. Heavy rainfall can lead to strong stratification, weaken turbulence, and suppress the upward transport of nutrients [23]. As a result, during the austral summer, Chl-a bloom is more likely to occur due to weak precipitation. In the study of Thompson et al. on the global precipitation problem, they concluded that phytoplankton responded more positively to increased precipitation during summer rather than winter [24]. Increased precipitation in winter was likely to reduce Chl-a, diatoms and chrysophytes, whereas increasing precipitation in summer was likely to increase Chl-a and favor chlorophytes.

The Chl-a bloom phenomenon can be seen more visually from Table 2, which contrasts the enhanced horizontal Chl-a transport, with a 40-fold increase in Chl-a flux at the western boundary of box B. More differences in the marine environment are shown between austral summer and austral winter, which demonstrate that austral summer is more suitable for Chl-a bloom.

**Table 2:** Comparison of Chl-a data, Chl-a flux, current flow, PAR, and precipitation data in box B.

season	Average Chl-a concentrations(μmol·L^-1^)	Average Chl-a flux at the western boundary(μmol·L^-1^)	Current strength(m·s^-1^)	PAR (Einstein m^2^·day^-1^)	Precipitation (mm·day^-1^)
austral summer	0.12	0.04	0.56	46.66	10.57
austral winter	0.03	-0.001	0.45	8.73	22.57
increase multiplier	4.30	44.32	1.24	5.343	0.49

## Discussion

### The influence of Agulhas

From Figs 6 and 7, it can be found that the path of surface Chl-a horizontal transport passed from the northeast to the southeast of Box A and finally transports eastward into Box B. When the ring is not dislodged (Fig 4A–4C), the large retroflection region can be an obstacle to the horizontal southeastward transport of Chl-a. Delcroix et al. mentioned that vortex horizontal transport can occur in two ways [25]; one of them is when transmitting over shorter distances, displacing fluid around their periphery while rotating. In Fig 4E and 4F, the Agulhas ring is detached in the form of an eddy, where Chl-a is transferred horizontally around the main axis of the retroflection. As the Agulhas ring flows in the opposite direction to the retroflection region, the resulting upwelling between the two may be responsible for the low loss of Chl-a after long periods of horizontal transport. In addition, the flow of Agulhas out of the African coast became larger in December 2013 and January 2014 (Fig 5), which also contributed to the horizontal southeastward transport of Chl-a.

In addition, the coastal nutrient-rich water from the Agulhas Current was found to be advection by Agulhas Return Current along its pathway [26]. The Chl-a bloom in this region is thought to be an effect of thermal fronts and local bathymetry. As the turbulence-induced by frontal variation generates eddies along the Agulhas Return Current path, making the condition favorable for Chl-a bloom. Interesting, the position they researched is close to Box B. Therefore, the Chl-a bloom in box B may be affected by the same factors.

### The role of stratification

**Fig 11:**
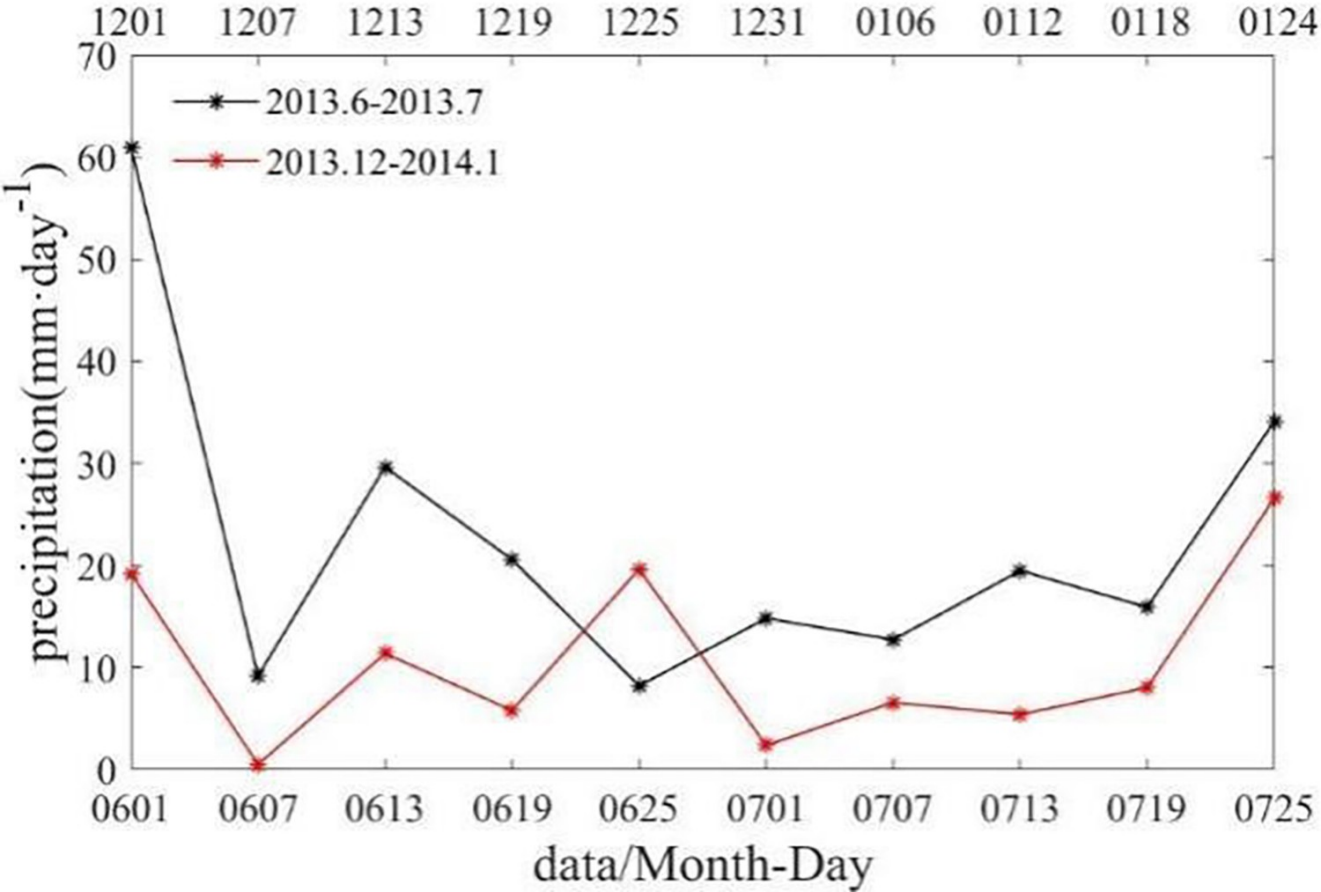
Time series of spatially averaged precipitation in Box B in June and July 2013 (black line), December 2013 and January 2014 (red line).

Some studies show that as the mixed layer deepens, the upper ocean stratification weakens, which promotes the accumulation of phytoplankton in the water [27,28]. In Fig 11, on January 10, 2014, the depth of the thermocline was located at 23.85 m, and the Fourier frequency was 0.0296 s^-1^. By January 20, 2014, the depth of the thermocline deepened to 39.68 m, and the Fourier frequency decreased to 0.0187 s^-1^. Combined with Fig 6, the concentration of nitrate was higher on the south side than on the north side of the Agulhas Current. In addition, frequent vertical mixing along the east coast of South Africa also brings nutrient-rich central South Indian waters into the surface coastal waters of the Agulhas Current system [29,30]. The weakened thermocline can uplift nutrients from deep water to the surface through upwelling, thus contributing to the Chl-a bloom. In their study of Agulhas Current from 2003 to 2018, Behera et al. concluded that not only the east-west movement of the Agulhas retroflection region had seasonal characteristics, but also the changes of SST and mixed layer depth had similar seasonal characteristics [26]. The mixed layer is deep in winter and shallow in summer. Therefore, the deepening of the mixed layer occurs from summer to winter.

### The influence of ocean eddies

**Fig 12:**
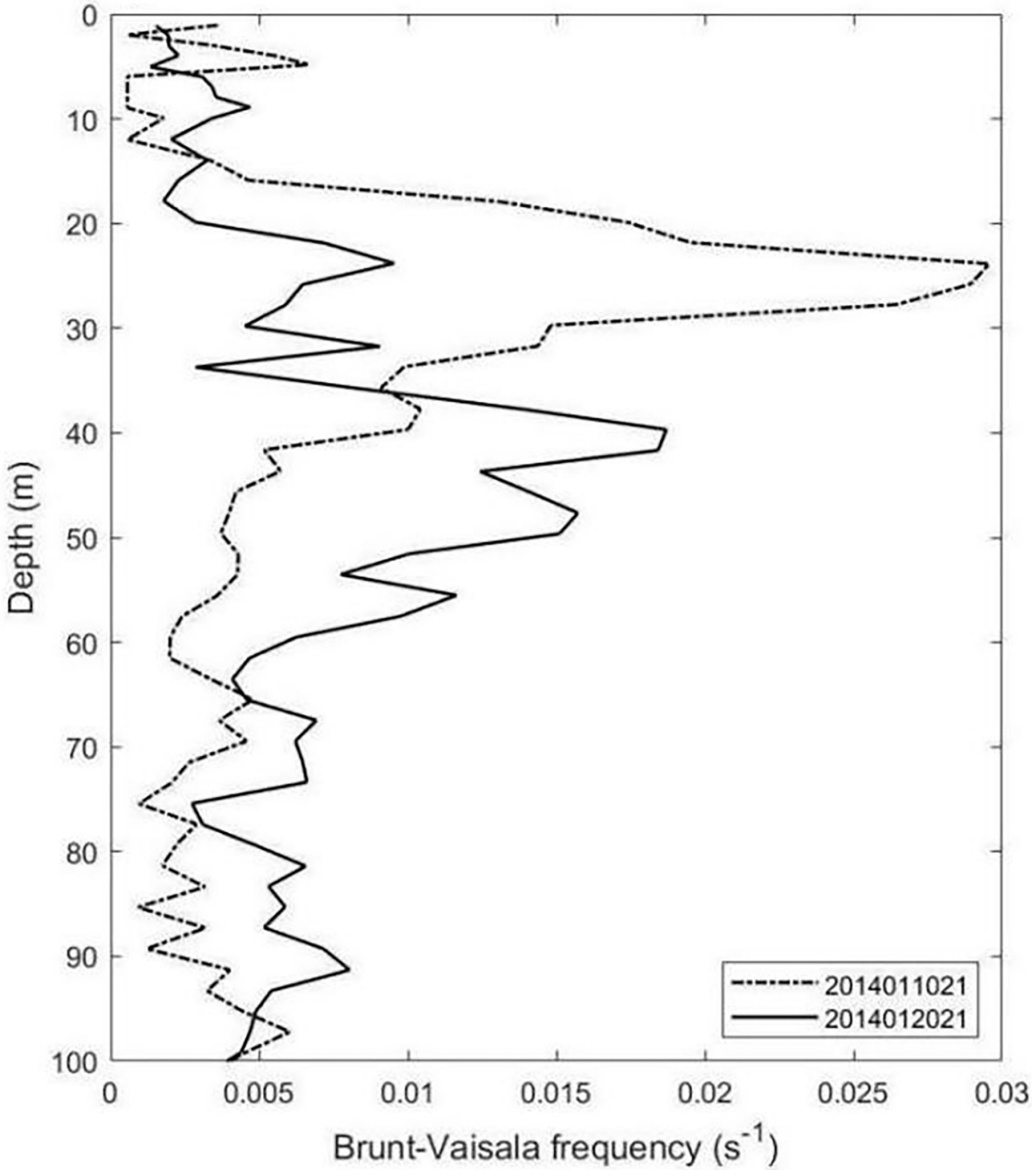
Brunt-Vaisala frequency from Argo float in January 2014.

**Fig 13:**
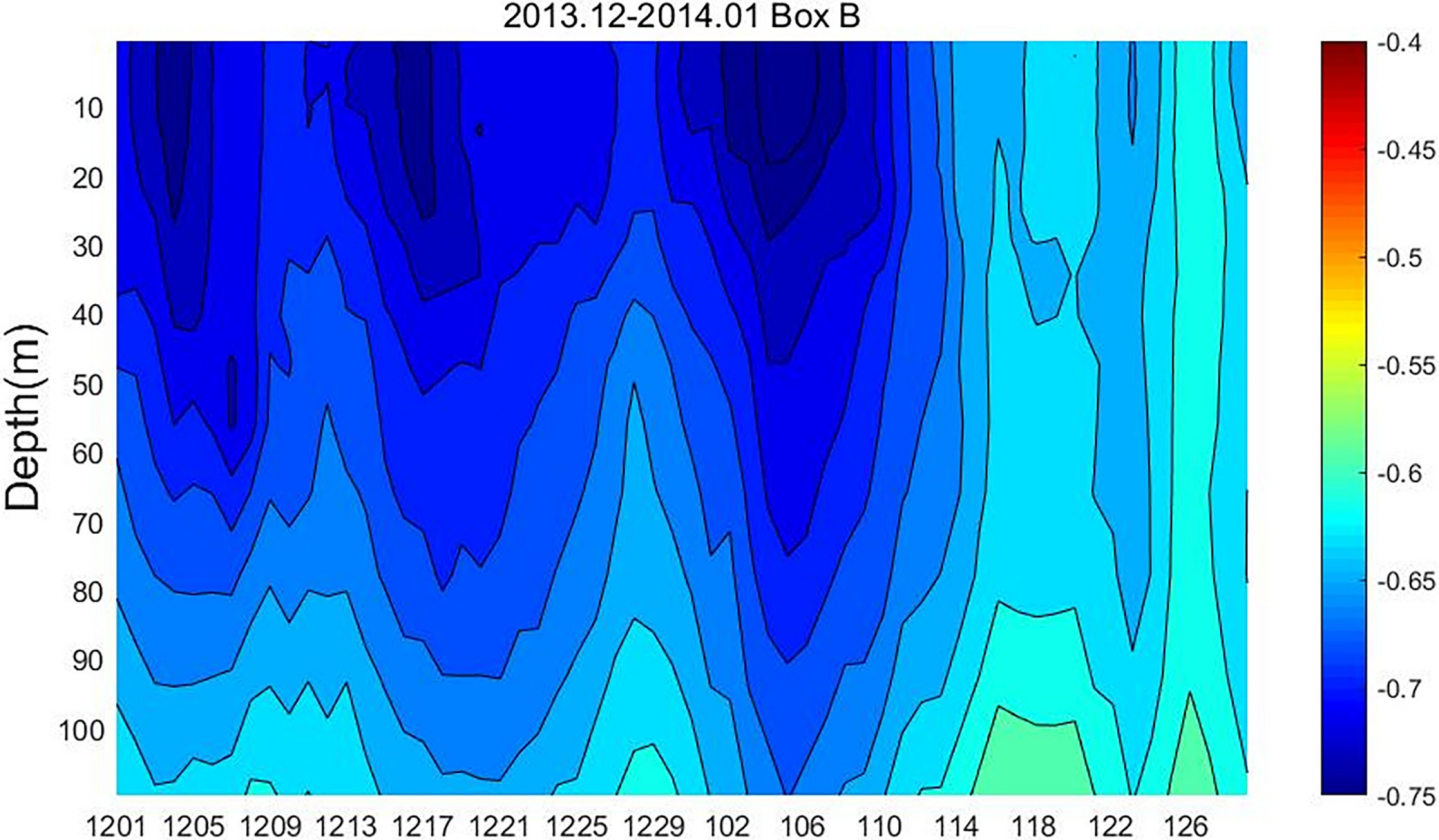
Time series of spatially averaged vorticity in Box B from December 2013 to January 2014.

**Fig 14:**
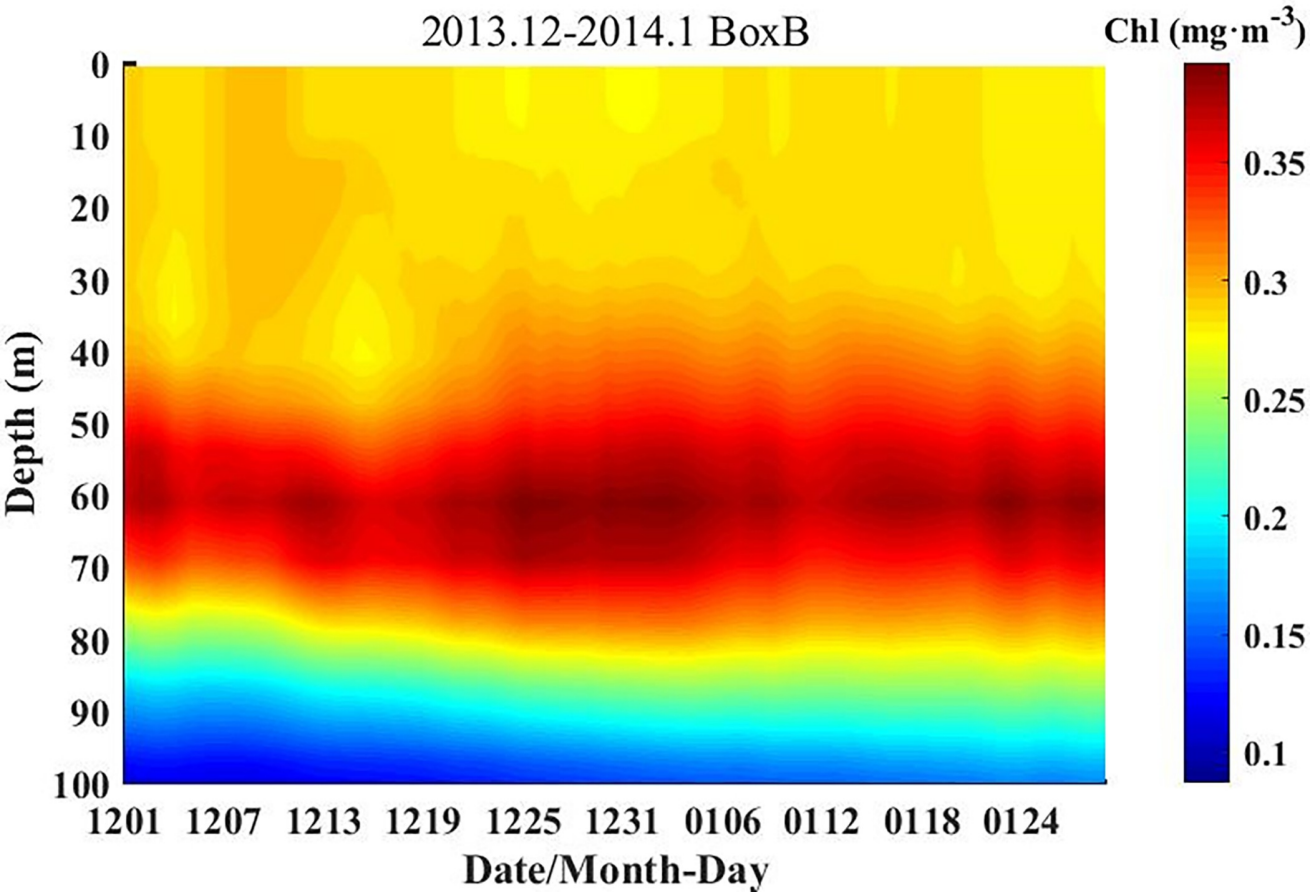
Time series of spatially averaged Chl-a in Box B from December 2013 to January 2014.

Gaube et al. suggested that when eddies affect the upper ocean through vertical and horizontal advection, the degree of influence depends on the polarity of the eddies (cyclones and anticyclones) and the state at the core of the eddies at the time of formation [13]. Sun et al. also found that both anticyclones and cyclones experience an abrupt deepening of energy at 38°S in the study area, but the cyclones are slightly weaker than the anticyclones [31]. A time series of spatially averaged vorticity in Box B from December 2013 to January 2014 is shown in Fig 12. A time series of spatially averaged Chl-a in Box B from December 2013 to January 2014 is shown in Fig 13. It can be seen that in December 2013 and January 2014, the vortex intensity in Box B was maintained at a higher intensity within 100 m; in particular, the stronger intensity with an oscillation appeared in the upper water. This phenomenon can be discussed in conjunction with Fig 14, which shows a tendency for Chl-a to uplift into surface layers from a depth of 60 m. The cyclonic eddies can influence the distribution of chlorophyll by uplifting the nutrient in the euphotic zone [26]. In the Southern Hemisphere, cyclonic eddies are more likely to produce upwelling and contribute to phytoplankton aggregation due to the rise in surface dispersion and eddy cores [32]. A similar phenomenon has been found in the Southeast Indian Ocean by [33], where chlorophyll bloom occur in the nutrient-poor Southeast Indian Ocean under the combined effect of eddies and winter mixing. Among them, eddy-Ekman pumping promoted the growth of phytoplankton in the subsurface Chl-a maximum layer. In addition, a study by Tan et al. in southern India peninsular found that the presence of vortices contributes to Chl-a bloom [14].

In the southeast Indian Ocean, the vortex carrying Chl-a was observed to propagate 600 km by He et al., which was the joint action of winter mixing and eddy-Ekman pumping [33]. Eddy-Ekman pumping can usually be produced by the interaction of surface winds and vortices [33]. The Agulhas retroflection region evaluated in this study is located directly in the westerly wind belt with strong cyclonic and anticyclonic vortices. In Fig 2, in Box B, cyclonic vortices can be observed, and many small anticyclonic vortices can be seen. From the studies of Early et al. in other parts of the Southern Indian Ocean, it can also be seen that there is a Chl-a bloom within the anticyclonic eddies due to eddy-Ekman pumping [34]. The high intensity of the cyclonic vortex in Box B and the resulting eddy-Ekman pumping under the wind stress of the westerly zone lift nutrients from deep water to the surface, which may also be an important cause of Chl-a bloom on the water surface in this region.

## Conclusions

In this study, the mechanism of Chl-a bloom on the south side of the Agulhas Current during December 2013 and January 2014 was studied using satellite remote sensing data, reanalysis data and Argo data. The conclusions are as follows.

1. The movement of the transitions of the Agulhas retroflection region has a seasonal character, moving westward in June, July 2013 (austral summer) and eastward in December 2013 and January 2014 (austral winter).
2. A Chl-a bloom was observed on the south side of the Agulhas Current in December 2013 and January 2014, which may be influenced by the eastward shift of the retroflection. Without the complex eddies in the retroflection region, the horizontal transport of Chl-a was enhanced along the south side of the Agulhas Current.
3. With sufficient light, a small amount of precipitation, a deepened thermocline, a reduced Fourier frequency, strong cyclonic eddies with upwelling and sufficient nutrients during December 2013 and January 2014, Chl-a bloom appeared on the south side of the Agulhas Current.
